# Synthesis of ZnO Nanoparticles by *Bacillus subtilis* for Efficient Photocatalytic Degradation of Cyanide

**DOI:** 10.3390/nano15070501

**Published:** 2025-03-26

**Authors:** Alberto Bacilio Quispe Cohaila, Gabriela de Lourdes Fora Quispe, Wilson Orlando Lanchipa Ramos, César Julio Cáceda Quiroz, Rocío María Tamayo Calderón, Jesús Plácido Medina Salas, Saravanan Rajendran, Elisban Juani Sacari Sacari

**Affiliations:** 1 Grupo de Investigación GIMAECC, Facultad de Ingeniería, Universidad Nacional Jorge Basadre Grohmann, Avenida Miraflores S/N, Ciudad Universitaria, Tacna 23003, Peru; 2 Laboratorio de Generación y Almacenamiento de Hidrogeno, Facultad de Ingeniería, Universidad Nacional Jorge Basadre Grohmann, Avenida Miraflores S/N, Ciudad Universitaria, Tacna 23003, Peru; 3 Laboratorio de Biorremediación, Facultad de Ciencias, Universidad Nacional Jorge Basadre Grohmann, Avenida Miraflores S/N, Ciudad Universitaria, Tacna 23003, Peru; 4 Facultad de Ciencias, Universidad Nacional de Ingeniería, Av. Túpac Amaru 210, Lima 15333, Peru; 5 Laboratorio de Compuestos, Departamento de Ingeniería de Materiales, Facultad de Ingeniería de Procesos, Universidad Nacional de San Agustín de Arequipa, Arequipa 04001, Peru; 6 Laboratorio de Nanotecnología, Facultad de Ingeniería, Universidad Nacional Jorge Basadre Grohmann, Avenida Miraflores S/N, Ciudad Universitaria, Tacna 23003, Peru; 7 Instituto de Alta Investigación, Universidad de Tarapacá, Arica 1000000, Chile

**Keywords:** zinc oxide nanoparticles, ZnO, UV photocatalysis, biosynthesis, *Bacillus subtilis*, cyanide degradation, green synthesis, biogenic nanoparticles

## Abstract

This study presents a sustainable and scalable biosynthesis method for zinc oxide (ZnO) nanoparticles using *Bacillus subtilis*, focusing on their application in photocatalytic cyanide degradation in aqueous solutions. The bacterial strain was molecularly identified through 16S rRNA gene sequencing and phylogenetic analysis. The optimized biosynthesis process yielded crystalline ZnO nanoparticles in the zincite phase with an average size of 21.87 ± 5.84 nm and a specific surface area of 27.02 ± 0.13 m^2^/g. Comprehensive characterization confirmed the formation of high-purity hexagonal ZnO (space group P63mc) with a bandgap of 3.20 eV. Photocatalytic tests under UV irradiation demonstrated efficient concentration-dependent cyanide degradation, achieving 75.5% removal at 100 ppm and 65.8% at 500 ppm within 180 min using 1.0 g/L ZnO loading. The degradation kinetics followed a pseudo-first-order model with rate constants ranging from 6.64 × 10^−3^ to 3.98 × 10^−3^ min^−1^. The enhanced photocatalytic performance is attributed to the optimal crystallite size, high surface area, and surface defects identified through a microscopic analysis. These results establish biosynthesized ZnO nanoparticles as promising eco-friendly photocatalysts for industrial wastewater treatment.

## 1. Introduction

Cyanide is a highly toxic contaminant present in effluents from various industries, especially in gold and silver mining. Its presence in water bodies represents a serious risk to human health and aquatic ecosystems, even at low concentrations. The toxicity of cyanide is due to its ability to inhibit cellular respiration by strongly binding to the enzyme cytochrome c oxidase, blocking electron transport in the mitochondrial respiratory chain [1]. This causes a lethal decrease in ATP in cells, which can lead to the rapid death of exposed organisms. The environmental impact of cyanide contamination extends beyond acute toxicity, affecting biodiversity and ecosystem functions in both aquatic and terrestrial environments [2].

Conventional methods for cyanide degradation, as shown in Table 1, such as alkaline oxidation or the INCO process, present significant limitations that hinder their effective and sustainable implementation. These methods often involve high operating costs due to the use of large quantities of expensive chemical reagents. In addition, their efficiency is limited, especially for low cyanide concentrations (<50 mg/L), making them unsuitable for treating diluted effluents. Another significant problem is the generation of toxic by-products, such as cyanate (CON^−^), which, although less toxic than cyanide, remains harmful to the environment. Many of these processes also require extreme operating conditions, such as significant pH adjustments, which complicates their implementation and increases costs. Finally, the disposal of sludge generated in some of these processes can be problematic, as it may contain toxic compounds that require special handling [3]. The limitations of these conventional methods have been extensively documented in recent reviews, highlighting the need for more efficient and sustainable alternatives [4,5,6].

In this context, there is an urgent need to develop more efficient, economical, and environmentally friendly technologies for the treatment of cyanide-contaminated effluents. Advanced oxidation processes (AOPs), particularly heterogeneous photocatalysis with nanostructured semiconductors, have emerged as a promising alternative to address these limitations. AOPs are characterized by the production of highly reactive oxidizing species, primarily hydroxyl radicals (•OH), which can rapidly degrade a wide range of organic and inorganic pollutants [12].

Among the various semiconductor materials, nanostructured zinc oxide (ZnO) presents characteristics that make it especially interesting for photocatalytic applications. These include a wide bandgap (3.37 eV) that allows efficient absorption of UV light, a high redox potential of photogenerated electron–hole pairs, chemical stability over a wide pH range, low toxicity, and good biocompatibility. Additionally, there is the possibility of synthesizing it in various nanostructures with high surface area, which enhances its catalytic activity [13].

The photocatalytic properties of ZnO nanoparticles have been extensively studied for the degradation of various pollutants, including dyes, pharmaceuticals, and pesticides [14,15]. Their efficiency is largely determined by synthesis methods, particle size, dopants [16], and surface defects, which modulate charge carrier dynamics and reactive oxygen species generation. Notably, zinc oxide synthesized via solvolysis in different alcohols has exhibited enhanced photocatalytic activity, demonstrating the material’s potential for environmental remediation [17].

However, the application of ZnO nanoparticles for cyanide degradation has been less explored, despite the potential for efficient removal of this highly toxic compound. Recent studies have shown promising results in the use of ZnO nanoparticles for photocatalytic cyanide degradation, demonstrating high removal efficiencies under optimized conditions [18,19]. However, the conventional synthesis of ZnO nanoparticles often involves the use of toxic chemical precursors and severe reaction conditions, which can partially counteract their environmental benefits. In this sense, biosynthesis mediated by microorganisms emerges as a greener and more sustainable alternative. Green synthesis methods have gained significant attention in recent years due to their potential to produce nanomaterials with controlled properties while minimizing environmental impacts [20,21]. Nevertheless, exhaustive research is still required to optimize these biosynthesis processes and evaluate the efficacy of the resulting materials in specific applications such as cyanide degradation.

The use of bacteria for the biosynthesis of metal oxide nanoparticles, including ZnO, has shown great promise due to the ability of microorganisms to interact with metal ions and mediate their transformation into nanostructures [22]. *Bacillus subtilis*, in particular, has been identified as an excellent candidate for nanoparticle biosynthesis due to its robust nature, well-characterized genetics, and ability to produce a wide range of metabolites that can act as reducing and capping agents [23]. This research project focuses precisely on addressing these challenges, seeking to develop an efficient and sustainable process for the photocatalytic degradation of cyanide using biosynthesized ZnO nanoparticles. The use of *Bacillus subtilis* as a biological mediator for the synthesis of ZnO nanoparticles offers several advantages, including the use of a non-pathogenic and widely available organism, as well as the potential for controlling nanoparticle properties through the optimization of culture and synthesis conditions [24].

This study aims to develop and optimize a green biosynthesis method for ZnO nanoparticles using *Bacillus subtilis*. It focuses on characterizing the nanoparticles’ structural, morphological, and optical properties and evaluating their photocatalytic efficiency in cyanide degradation under UV light. This research seeks to promote sustainable and efficient water treatment technologies, particularly in the context of environmental remediation. The findings could have significant implications for treating industrial effluents and advancing environmental nanotechnology.

## 2. Materials and Methods

### 2.1. Materials

All materials and reagents utilized in this study were obtained from Merck (Darmstadt, Germany) and were of analytical-grade purity. These included zinc acetate dihydrate (Zn(CH_3_COO)_2_·2H_2_O, ≥99.5%), sodium bicarbonate (NaHCO_3_, ≥99.7%), Brain Heart Infusion (BHI) broth, nutrient agar, nutrient broth, sodium cyanide (NaCN, ≥95%), silver nitrate (AgNO_3_, ≥99.8%), potassium iodide (KI, ≥99.5%), and tert-butanol (C_4_H_10_O, ≥99.5%). All solutions were prepared using sterile ultrapure water (18.2 MΩ·cm at 25 °C) obtained from a type I water purification system (Arium Mini, Sartorius AG, Göttingen, Germany).

### 2.2. Biogenic Synthesis of ZnO Nanoparticles

The biosynthesis of ZnO nanoparticles was conducted using *Bacillus subtilis*, previously isolated and characterized by Cáceda et al. [9], from the research strain collection of the Bioremediation Laboratory at Jorge Basadre Grohmann National University. The procedure of biogenic synthesis of the ZnO nanoparticles was adapted from Tripathy et al. [25], with modifications to optimize the process for large-scale production.

**Figure 1:**
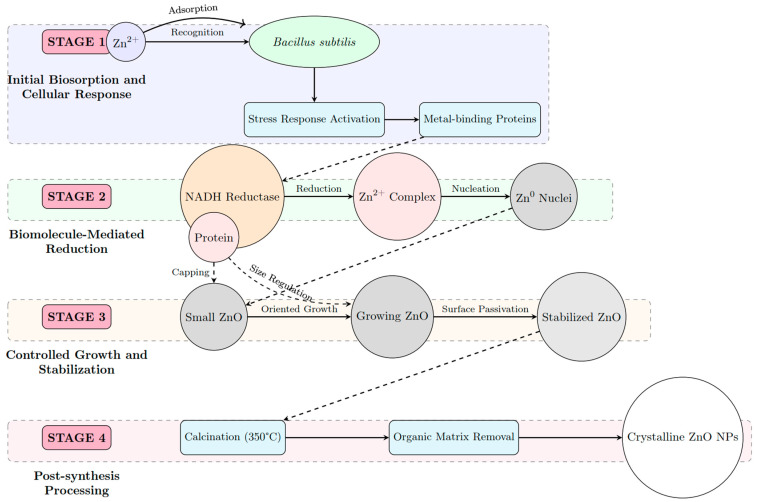
Schematic representation of biogenic synthesis of ZnO NPs.

The biogenic synthesis of ZnO nanoparticles using *Bacillus subtilis* proceeds through a complex, multi-step mechanism that leverages the natural biochemical processes of the microorganism as described in Figure 1. Initially, Zn^2+^ ions from zinc acetate dihydrate are recognized by cell-surface proteins, triggering a stress response that upregulates metal-binding proteins and defense mechanisms. This biosorption phase is followed by biomolecule-mediated reduction, where NADH-dependent reductases and other enzymes secreted by *B. subtilis* facilitate the reduction of Zn^2+^ to Zn^0^, forming initial nuclei. The controlled growth phase then begins, characterized by the oriented attachment of zinc atoms and crystal growth, while extracellular proteins simultaneously act as capping agents that regulate particle size and prevent agglomeration [26]. These proteins bind to specific crystal facets, stabilizing the growing nanoparticles and conferring a characteristic hexagonal wurtzite structure. The final phase involves post-synthesis processing through calcination, which removes the organic matrix while preserving the crystalline integrity, resulting in high-purity ZnO nanoparticles [27]. This biogenic route offers significant advantages over conventional chemical methods, including milder reaction conditions, environmentally friendly precursors, and enhanced photocatalytic activity due to the unique surface properties imparted by the biological synthesis process.

Initially, the *B. subtilis* strain was reactivated in Brain Heart Infusion (BHI) broth for 24 h at 35 °C. The culture was then streaked on nutrient agar plates and incubated for an additional 24 h at 35 °C to obtain fresh colonies. Gram staining confirmed the presence of Gram-positive, rod-shaped bacteria with rounded edges and endospores, typical of *B. subtilis*.

For nanoparticle synthesis, *B. subtilis* was cultured in nutrient broth at 35 °C and 180 rpm for 48 h. The bacterial pellet was harvested by centrifugation at 8000 rpm for 10 min. ZnO nanoparticle biosynthesis was initiated by inoculating 2 g of the bacterial pellet into a precursor solution composed of 375 mL of 0.2 M zinc acetate dihydrate and 375 mL of 0.5 M sodium bicarbonate in sterile ultrapure water. This mixture was incubated at 35 °C and 170 rpm for 72 h to facilitate the interaction between bacteria and precursors, leading to ZnO nanoparticle formation. All measurements were conducted with strict adherence to metrological principles using a calibrated analytical balance (Sartorius ENTRIS224-1S) with a resolution and standard deviation of 0.1 mg.

Post-incubation, the solution underwent centrifugation at 8000 rpm for 10 min. The precipitate was subjected to six cycles of washing with sterile ultrapure water and centrifugation to eliminate impurities and residual metabolites. The washed precipitate was dried at 60 °C for 18 h and analyzed by thermogravimetric analysis and differential scanning calorimetry. Crystallization was achieved by finely grinding the dried product using an agate mortar and calcining it at 350 °C for 2 h in a porcelain crucible. A final grinding step was performed to de-agglomerate the ZnO nanoparticles formed during calcination, ensuring a fine powder suitable for further characterization and application.

While the molecular identification was previously reported by Cáceda et al. [9], this study further analyzed the phylogenetic relationships of the strain. A phylogenetic tree was constructed using the Maximum Likelihood algorithm based on the 16S rRNA gene sequence (GenBank accession number OR505001.1). This additional analysis provides a more comprehensive understanding of the bacterial strain used in the biosynthesis process.

### 2.3. Characterization of Biosynthesized ZnO Nanoparticles

The characterization of the biosynthesized ZnO nanoparticles was carried out using a range of analytical techniques. Thermogravimetric analysis (TGA) and differential scanning calorimetry (DSC) were performed on pre-calcination samples using an SDT650 instrument (TA Instruments, New Castle, DE, USA) under nitrogen flow (100 mL/min) with a heating rate of 20 °C/min. X-ray diffraction (XRD) analysis was conducted on both pre- and post-calcination samples using a PANalytical Aeris Research diffractometer with Cu K_α_ radiation at 40 kV and 15 mA (Malvern Panalytical Ltd., Almedo, The Netherlands). Optical properties were studied using a ThermoScientific Evolution 220 UV–visible spectrophotometer (Thermo Scientific Co., Ltd., Waltham, DE, USA). The vibrational spectra were obtained using a Bruker Invenio R spectrometer (Ettlingen, Germany) with attenuated total reflectance Fourier-transform infrared (ATR-FTIR) spectroscopy over the 400–4000 cm^−1^ range. Photoluminescence spectra were acquired using a FluoroMax Plus spectrometer (Horiba Scientific, Irvine, CA, USA). Transmission electron microscopy (TEM) was performed using a Thermo Scientific Talos 200i microscope operating at 200 kV (Thermo Scientific Co., Eindhoven, The Netherlands), while scanning electron microscopy (SEM) utilized a Thermo Scientific Quattro S microscope at 30 kV under high vacuum (Thermo Scientific Co., Eindhoven, The Netherlands). Specific surface area was determined by the Brunauer–Emmett–Teller (BET) method using a Micrometrics Gemini VII physisorption analyzer (Micromeritics Instrument Co., Norcross, GA, USA).

### 2.4. Photocatalytic Assessment of ZnO for Cyanide Degradation

The photocatalytic performance of the biosynthesized ZnO nanoparticles for cyanide degradation was evaluated using a custom-designed 1 L quartz photocatalytic reactor. A series of sodium cyanide (NaCN) solutions with concentrations ranging from 100 to 500 ppm were prepared in the reactor vessel, with the pH carefully adjusted to 10 to minimize CN^−^ volatilization. The biosynthesized ZnO nanoparticles were added at two different concentrations (0.5 and 1 mg/mL) to assess the effect of catalyst loading on degradation efficiency. The reactor was equipped with a 254 nm UV light source and 11W (HNS S 11 W G23, Osram, Germany), introduced via a quartz tube, to initiate the photocatalytic process. Throughout the 3 h reaction period, the solution was continuously stirred at 500 rpm and maintained at 25 °C to ensure uniform catalyst dispersion and consistent reaction conditions. To monitor the progression of cyanide degradation, aliquots were extracted at regular intervals and analyzed using a titrimetric method based on Standard Methods 4500-CN-D. This analytical approach, which relies on the reaction between silver nitrate (AgNO_3_) and cyanide ions (CN^−^) to form a soluble silver cyanide complex (Ag(CN)), provided a reliable means of quantifying the remaining cyanide concentration. The titration endpoint was visually determined by the appearance of a faint yellow color upon the addition of a potassium iodide (KI) indicator. The volume of AgNO_3_ consumed in the titration was used to calculate the free cyanide concentration, with 1 mL of AgNO_3_ titration consumption equivalent to 20 ppm of CN^−^. The cyanide degradation efficiency (DE) was then calculated using the equation

$$DE=\frac{\left(C_{i}-C_{r}\right)}{C_{i}}\cdot 100$$

where C_i_ represents the initial cyanide concentration and C_r_ the residual concentration after photocatalytic treatment.

The reusability of biosynthesized ZnO nanoparticles is a critical factor for their practical application in industrial wastewater treatment systems. To evaluate this aspect, the photocatalyst was recovered after each degradation cycle through centrifugation at 6000 rpm for 10 min, followed by washing with deionized water and drying at 60 °C for 2 h. Successive photocatalytic cycles were conducted under identical conditions (0.5 and 1.0 g/L catalyst loading, 100 ppm initial cyanide concentration, pH 10, and UV irradiation at 254 nm).

## 3. Results

### 3.1. Phylogenetic Analysis of the Bacterial Strain

The bacterial strain used for ZnO nanoparticle biosynthesis, which was previously isolated by Cáceda et al. [9], underwent further phylogenetic analysis in this study. The 16S rRNA gene sequence (1538 base pairs) of this strain, originally identified by Cáceda et al., was reanalyzed and compared with the NCBI GenBank database, confirming 100% identity with four previously reported *B. subtilis* sequences (accession numbers: OL636042.1, KR967391.1, EU047884.1, and CP025941.1).

**Figure 2:**
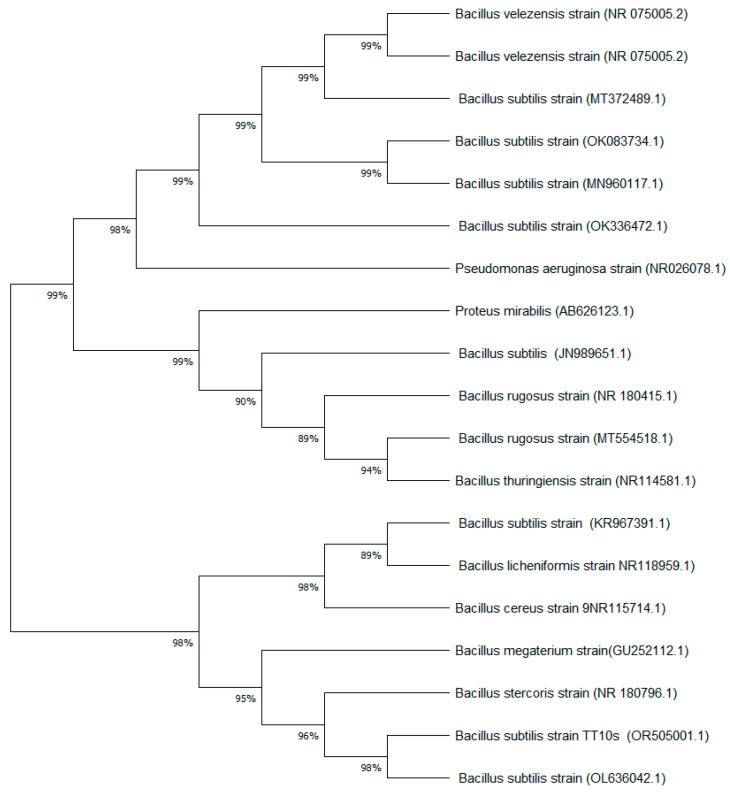
Phylogenetic tree of *Bacillus subtilis*.

To further elucidate the taxonomic positioning of the strain, we conducted a phylogenetic analysis using the Maximum Likelihood algorithm. This analysis corroborated the strain’s classification within the *B. subtilis* clade, as illustrated in Figure 2. Notably, our strain exhibited a particularly close relationship with the sequence ID OL636042.1. The 16S rRNA gene sequence used in this analysis is accessible in GenBank under the accession number OR505001.1.

The phylogenetic analysis not only confirmed the taxonomic identity of the strain but also revealed its close relationship with *B. subtilis* strains previously reported for nanoparticle synthesis. This genetic proximity suggests similar metabolic capabilities for metal oxide synthesis, particularly in terms of stress response mechanisms and metal-binding protein production. The confirmed strain identity provides a reliable foundation for the reproducibility of our biosynthesis method, as *B. subtilis* is known for its robust metabolic machinery and ability to produce diverse secondary metabolites that can act as both reducing and stabilizing agents in nanoparticle formation [28].

### 3.2. Thermal Analysis Results

**Figure 3:**
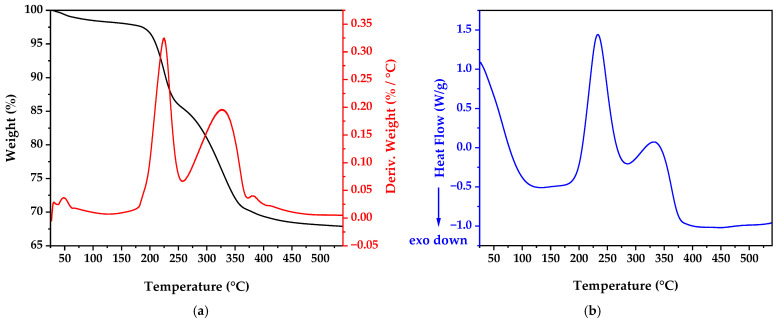
(**a**) TGA and DTA and (**b**) DSC results of the biosynthesized material.

The thermogravimetric analysis (TGA), derivative thermogravimetric analysis (dTG), and differential scanning calorimetry (DSC) results displayed in Figure 3 provide crucial information about the thermal behavior and phase transformations of the biosynthesized material before calcination. As shown in Figure 3a, the TGA profile revealed a multi-stage transformation process. Initially, in the range of 25 to 200 °C, a weight loss of approximately 3.5% was observed, attributable to the elimination of adsorbed water and volatile organic residues from the bacterial culture medium. The dTG curve in this region shows a small peak, confirming this as a distinct decomposition stage. Between 200 °C and 350 °C, a more pronounced weight loss (approximately 12%) occurred, corresponding to the decomposition of biomolecules and organic compounds that served as capping agents during nanoparticle formation [29]. This is evidenced by a prominent peak in the dTG curve centered around 230 °C. The third and most substantial weight loss stage (approximately 18%) occurred between 350 °C and 500 °C, likely due to the decomposition of zinc precursor residues and the crystallization of amorphous zinc species into ZnO, clearly visible as the largest peak in the dTG curve centered at approximately 320 °C. The DSC curve (Figure 3b) correlates with these weight loss stages, showing initial exothermic events (downward peaks) in the first region (50–200 °C), followed by endothermic transitions (upward peaks) at 230 °C and 320 °C. The exothermic nature of the first stage could be attributed to crystallization processes or oxidation reactions occurring simultaneously with water evaporation, while the endothermic peaks correspond to the decomposition of organic material and precursor compounds. These thermal events guided the selection of the optimal calcination temperature (350 °C) to ensure complete removal of organic matter while maintaining the nanoscale characteristics of the particles. This temperature is notably lower than those typically reported for conventional chemical synthesis methods (400–600 °C) [30,31], highlighting the energy efficiency of our biosynthetic approach.

### 3.3. X-Ray Diffraction (XRD) Analysis After Calcination

**Figure 4:**
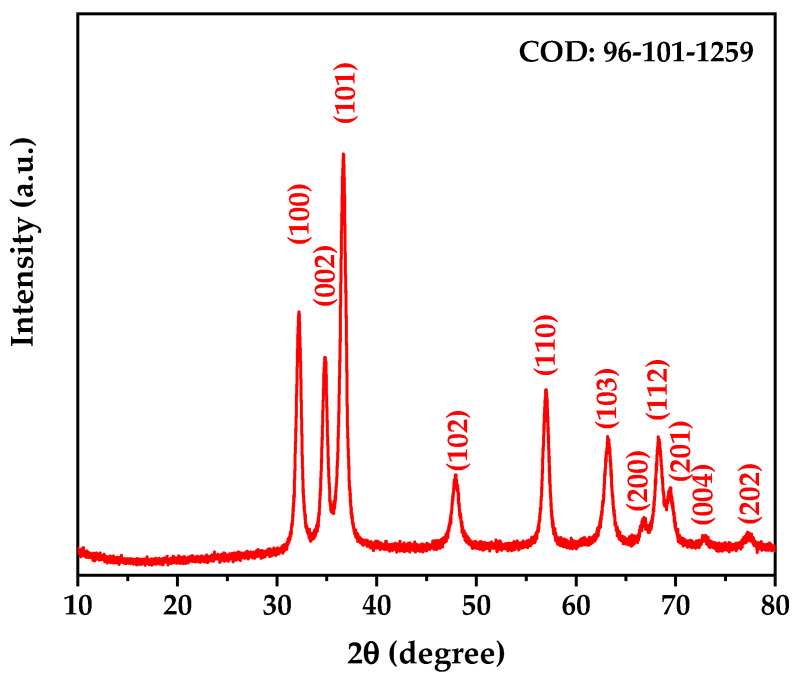
X-ray diffraction pattern of calcined ZnO nanoparticles.

The XRD pattern of the calcined ZnO nanoparticles (Figure 4) revealed a complete transformation in the crystal structure of the biosynthesized material, evidencing the successful formation of ZnO in its zincite phase. The observed diffraction peaks were characteristic of the crystalline structure of ZnO in the zincite phase, which was confirmed by correspondence with the crystallographic card COD 96-101-1259 [32].

Rietveld refinement was performed using PANalytical HighScore Plus software V.4.9, providing detailed information on the crystal structure of the synthesized ZnO. The results shown in Table 2 confirm the hexagonal structure with space group P63mc (space group number 186), characteristic of the zincite phase. The refined lattice parameters, a = b = 3.25168 Å and c = 5.21387 Å, with angles α = β = 90° and γ = 120°, are consistent with the expected values for high-purity ZnO. The calculated density of 5.65 g/cm^3^ is in excellent agreement with the theoretical value for crystalline ZnO.

Previous studies have established that crystallite size plays a crucial role in determining photocatalytic efficiency, with an optimal range typically between 10 and 50 nm [33,34]. This size range provides an ideal balance between surface area and crystal quality—small enough to offer abundant surface active sites while maintaining sufficient crystallinity to minimize defect-induced recombination centers [35,36]. Within this context, the crystallite size of our biosynthesized ZnO (14.18 nm, determined through Rietveld refinement) falls within this optimal range for photocatalytic applications.

The well-defined and narrow diffraction peaks observed in the XRD pattern (Figure 4) indicate high crystallinity of the obtained material. The relative intensity of the peaks, particularly the prominence of the (101) peak, is consistent with the formation of highly crystalline ZnO. The absence of any secondary phases or impurities confirms the complete transformation into pure ZnO at the selected calcination temperature. The low residual stress (0.004%) further indicates excellent structural stability, which is crucial for maintaining consistent photocatalytic performance over multiple cycles. The high goodness of fit (GOF) value from our Rietveld analysis further confirms the accuracy of our structural characterization.

### 3.4. Fourier-Transform Infrared Spectroscopy (FTIR) Analysis

**Figure 5:**
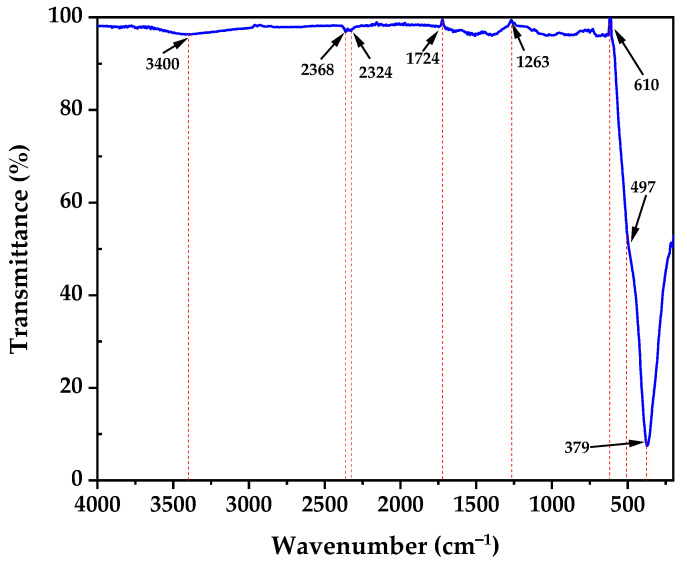
FTIR of ZnO nanoparticles.

The FTIR spectrum of the calcined ZnO nanoparticles displayed in Figure 5 reveals several characteristic bands that confirm the formation of zinc oxide in the zincite structure. A broad band centered around 3400 cm^−1^ is attributed to O-H stretching vibrations [37], likely from adsorbed water molecules on the nanoparticle surface. Bands at 2368 cm^−1^ and 2324 cm^−1^ are likely due to atmospheric CO_2_ present during measurement [38]. Peaks at 1724 cm^−1^ and 1263 cm^−1^ indicate the presence of C=O stretching vibrations and C-O-C anti-symmetric stretching vibrations, respectively [39]. Most significantly, the sharp peak at 610 cm^−1^ and the strong absorption band below 500 cm^−1^ (centered around 497 cm^−1^) are characteristic of Zn-O stretching vibrations in the ZnO lattice, strongly confirming the formation of ZnO nanoparticles in the zincite phase [40]. The presence of these strong Zn-O vibration bands provides compelling evidence for the successful synthesis of ZnO nanoparticles, while the weak organic bands suggest that the calcination process effectively removed most organic residues, resulting in relatively pure ZnO nanoparticles. The FTIR analysis not only confirmed the formation of ZnO but also provided insights into the surface chemistry of the nanoparticles. The weak organic bands suggest the presence of residual biomolecules that may act as surface modifiers, potentially enhancing the dispersion stability and photocatalytic activity of the nanoparticles. The results are consistent with the XRD results [41].

### 3.5. UV–Visible Spectroscopy

**Figure 6:**
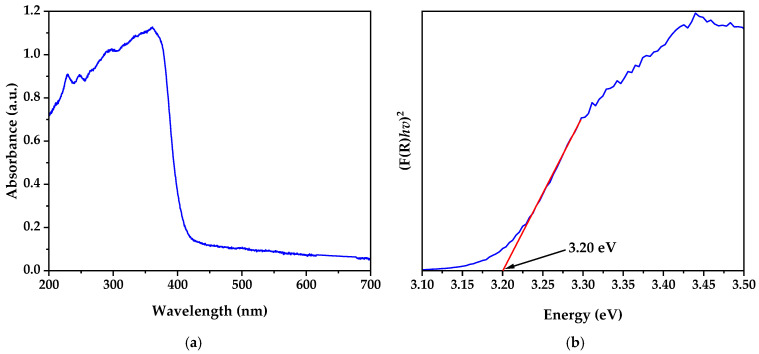
(**a**) UV–vis spectra and (**b**) Kubelka–Munk function plot.

The UV–visible absorption spectrum (Figure 6a) showed strong absorption in the ultraviolet region, with a pronounced absorption edge around 380–400 nm. This feature is typical of ZnO and is attributed to the electronic transition from the valence band to the conduction band [42].

The application of the Kubelka–Munk function and the Tauc plot (Figure 6b) allowed for a more precise determination of the material’s bandgap energy. The extrapolation of the linear part of the Tauc curve to the energy axis provided a bandgap value of 3.20 eV. This value is consistent with those reported for ZnO nanoparticles synthesized by chemical methods such as sol–gel (3.20 eV) and slightly higher than those prepared by room-temperature wet chemical methods (3.16 eV), as shown in Table 4. The bandgap energy slightly lower than the typical value of bulk ZnO (3.37 eV) could be attributed to the nanometric size of the particles and possible quantum confinement effects [43].

The pronounced shape of the absorption edge and the clarity of the transition in the Tauc plot indicate a narrow particle size distribution and high purity of the material [13,44]. This is consistent with the effectiveness of the biosynthesis method and subsequent heat treatment in producing well-crystallized and homogeneous ZnO nanoparticles.

### 3.6. BET Surface Area Analysis

**Figure 7:**
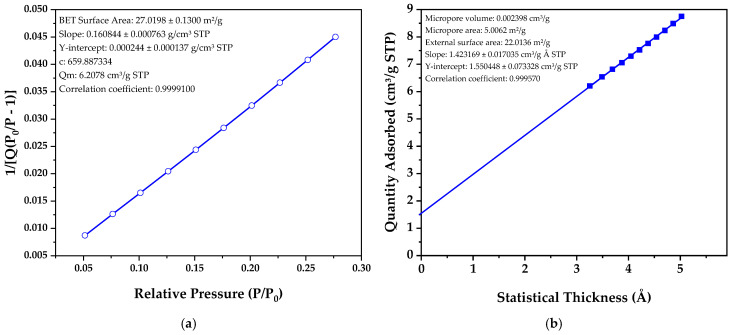
(**a**) Multi-point BET plot and (**b**) t-plot.

The nitrogen adsorption–desorption isotherms elucidated the morphological characteristics of the biosynthesized ZnO nanoparticles. The Brunauer–Emmett–Teller (BET) analysis (Figure 7a) demonstrated a specific surface area of 27.0198 ± 0.1300 m^2^/g, exhibiting a linear correlation coefficient of 0.9999100 within a relative pressure (P/P₀) domain of 0.05–0.30. The dimensionless BET interaction parameter (C) of 659.887334 indicates robust adsorbate–substrate interactions, characteristic of metal oxide interfaces. A t-plot analysis (Figure 7b) quantified the material porosity parameters, revealing a microporous surface area of 5.0062 m^2^/g with a corresponding micropore volume of 0.002398 cm^3^/g. The external surface area, determined to be 22.0136 m^2^/g, constitutes approximately 81.5% of the total surface area, indicating predominant accessibility through external crystallite facets rather than intraporous networks. These structural parameters, specifically the moderate specific surface area coupled with enhanced external surface accessibility, demonstrate optimal characteristics for heterogeneous photocatalysis [45] by facilitating enhanced mass transport phenomena and maximizing the availability of photocatalytically active sites during the cyanide degradation processes. The minimal micropore volume suggests reduced diffusion limitations for substrate molecules, thereby enhancing the overall photocatalytic efficiency of the system.

### 3.7. Scanning Electron Microscopy (SEM) Results

**Figure 8:**
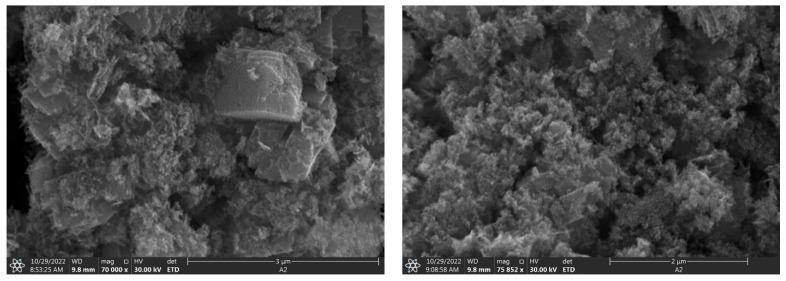
Scanning electron microscopy micrographs at different magnifications.

SEM images (Figure 8) revealed crucial information about the morphology and aggregation state of the synthesized ZnO nanoparticles. The nanoparticles presented an irregular morphology, which is common in biosynthesized materials due to the influence of biological processes on particle formation. A notable aspect is the tendency of nanoparticles to form agglomerates. These agglomerates, also irregular in shape, are composed of smaller primary particles. The formation of agglomerates is a frequent phenomenon in metal oxide nanoparticles and can be attributed to strong surface interactions between particles, possibly due to van der Waals forces or dipole–dipole interactions [46].

The energy-dispersive X-ray spectroscopy (EDX) analysis (Table S1) confirmed the elemental composition, showing the predominant presence of Zn and O with atomic percentages of 26.12% and 56.88%, respectively. Elemental mapping (Figure S1) further illustrated the homogeneous distribution of these elements throughout the nanoparticle structure.

### 3.8. Transmission Electron Microscopy (TEM) Results

**Figure 9:**
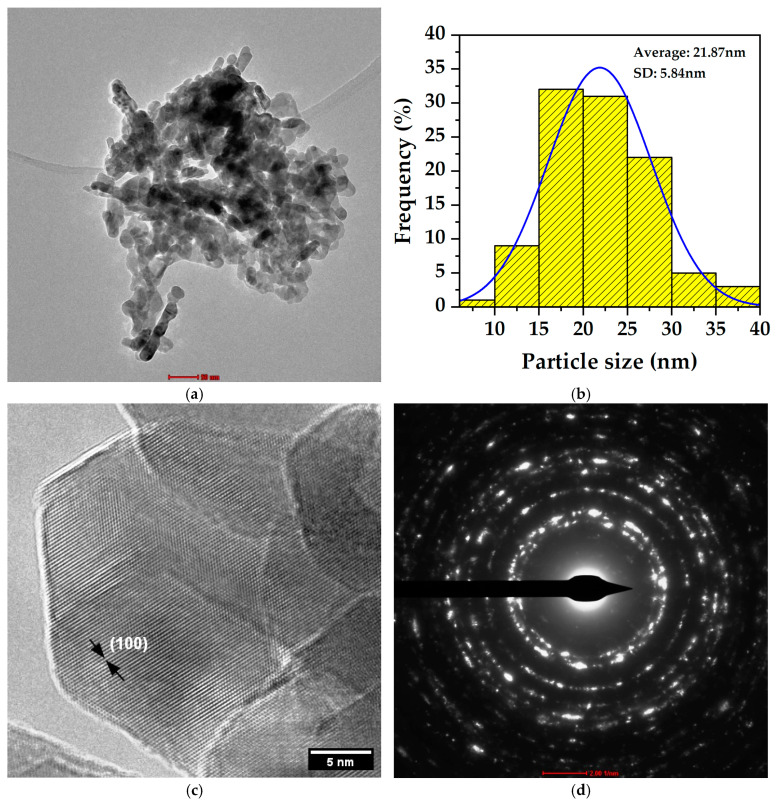
(**a**) ZnO nanoparticles, (**b**) Particle size distribution, (**c**) High-resolution image. (**d**) SAED pattern.

The TEM analysis provided detailed information about the nanoscale structure of the biosynthesized ZnO particles in the zincite phase. Low-resolution images (Figure 9a) revealed predominantly quasi-spherical nanoparticles with some irregularity in their contours and in some cases elongated shapes, typical characteristics of biosynthesized materials. The particle size distribution (Figure 9b) analysis showed an average size of 21.87 nm with a standard deviation of 5.84 nm, indicating a homogeneous population of nanoparticles with a relatively narrow distribution. This uniformity suggests good control of the biosynthesis process over nanoparticle growth.

The high-resolution TEM (HRTEM) images (Figure 9c) allowed observation of the crystalline planes of the ZnO nanoparticles in the zincite phase. The visible lattice fringes confirm the crystalline nature of the material, with a measured interplanar distance of 2.782 Å corresponding to the (100) plane of the hexagonal ZnO structure, validating the accuracy of the TEM measurement and confirming the crystallographic identity of the material as ZnO in the zincite phase.

The high-resolution TEM (HRTEM) images (Figure 9c) were specifically selected to provide clear visualization of the crystalline lattice structure, enabling the precise measurement of interplanar distances. While some particles in these HRTEM images may appear larger due to the focus on well-defined crystalline regions and possible particle overlap, they remain within the upper range of the size distribution shown in Figure 9b (21.87 ± 5.84 nm). It is important to note that the particle size distribution was determined by measuring over 100 individual nanoparticles to ensure statistical reliability, whereas the HRTEM analysis prioritizes regions with distinct lattice fringes for accurate crystallographic characterization.

The selected area electron diffraction (SAED) pattern (Figure 9d) exhibited well-defined concentric rings, characteristic of a polycrystalline material. The indexing of these rings coincides with the expected crystallographic planes for the zincite phase of ZnO. The most prominent rings correspond to interplanar distances of 2.80765 Å (100), 2.59400 Å (002), 2.46925 Å (101), 1.90530 Å (102), 1.62100 Å (110), and 1.47244 Å (103). These values perfectly agree with the provided diffraction data and unequivocally confirm the hexagonal structure of ZnO.

### 3.9. Photoluminescence Analysis

**Figure 10:**
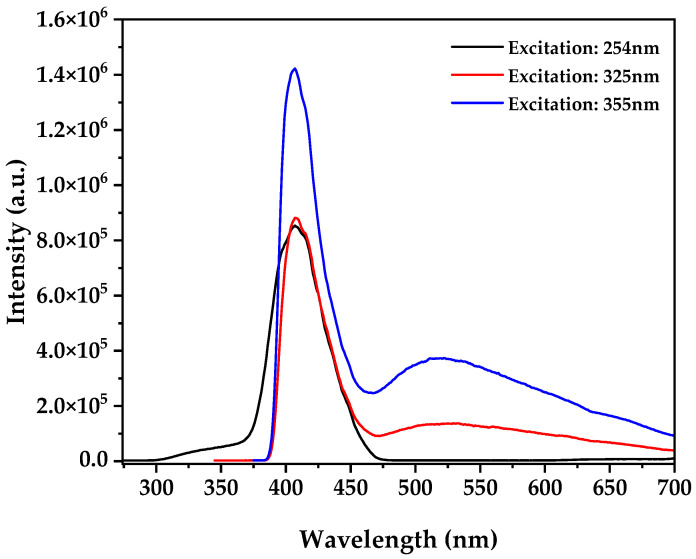
Photoluminescence spectra at different excitation wavelengths.

The photoluminescence spectra of the biosynthesized ZnO nanoparticles revealed distinctive optical characteristics (Figure 10). Three excitation wavelengths (254, 325, and 355 nm) were employed to comprehensively investigate the electronic states of the material. All spectra exhibited an intense near-band-edge (NBE) emission centered at ~400 nm, attributable to excitonic recombinations related to band-to-band transitions [47]. Excitation at 355 nm produced the highest NBE emission intensity (1.42 × 10^6^ a.u.), approximately 1.7 times higher than with 254 or 325 nm excitation. Additionally, an emission band in the visible region (450–650 nm) was observed with greater prominence under 355 nm excitation, indicative of specific structural defects: the green-blue emission (500–530 nm) is primarily associated with oxygen vacancies [48], while the blue emission (450–470 nm) corresponds to zinc interstitial defects [49]. The intensity ratio between the visible and NBE emissions (I_visible_/INBE = 0.30 for 355 nm excitation) suggests an optimized surface defect density that favors charge carrier separation and reduces recombination, thus contributing to the higher photocatalytic efficiency observed [50]. This balance between crystalline quality and beneficial defects is characteristic of our biosynthesis method and explains the enhanced photocatalytic performance in cyanide degradation [51].

### 3.10. Results of Cyanide Photocatalytic Degradation

**Figure 11:**
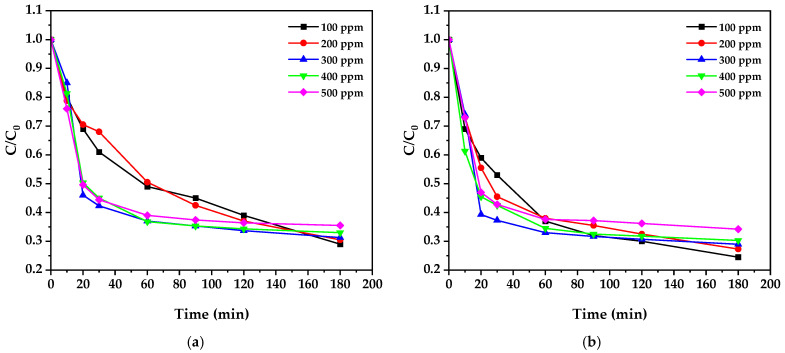
Normalized concentration (C/C_0_) for different initial cyanide concentrations with (**a**) 0.5 g/L and (**b**) 1 g/L ZnO, respectively.

The photocatalytic degradation of cyanide using biosynthesized ZnO nanoparticles was investigated under UV irradiation (λ = 254 nm). The experiments encompassed various initial cyanide concentrations (100–500 ppm) and catalyst loadings (0.5 and 1.0 g/L) to clarify the influence of these operational parameters on degradation efficiency (Figure 11). The temporal evolution of normalized cyanide concentration (C/C₀) exhibited characteristic decay profiles, with the most rapid degradation occurring within the first 30 min of irradiation, followed by a more gradual decrease until reaching equilibrium at approximately 180 min.

Analysis of the degradation profiles revealed a strong dependence on an initial cyanide concentration of 75. At lower concentrations (100 ppm), the process achieved higher removal efficiency, reaching 75.5% degradation with 1.0 g/L ZnO after 180 min. This efficiency decreased to 65.8% when the initial concentration was increased to 500 ppm under identical conditions, consistent with previous findings [52,53]. This behavior can be attributed to the saturation of available catalytic sites on the ZnO surface and increased competition for reactive oxygen species (ROS) at higher cyanide concentrations [54].

The influence of catalyst loading demonstrated an interesting concentration-dependent trend. At 100 ppm cyanide, increasing the ZnO loading from 0.5 to 1.0 g/L enhanced the degradation efficiency by 13.1%. However, this enhancement diminished significantly at higher cyanide concentrations, showing only a 2% improvement at 500 ppm. This phenomenon aligns with the findings of Farrokhi et al. [52], who proposed that an optimal catalyst-to-substrate ratio exists due to the competing effects of increased active sites and reduced light penetration at higher catalyst loadings.

As illustrated in Table 4, our biogenic ZnO nanoparticles demonstrate competitive photocatalytic performance when compared to various conventional synthesis methods. While the ZnO-BiOI heterojunction prepared by the precipitation method shows the highest degradation efficiency (97%) with the shortest reaction time (35 min), this system benefits from the synergistic effect of the heterojunction structure rather than ZnO properties alone. Among single-component ZnO catalysts, copper phthalocyanine-sensitized ZnO achieved 95% degradation but required twice the reaction time (360 min) compared to our biogenic approach. Notably, our biogenic ZnO at 1 g/L loading achieved a 75.5% degradation efficiency, outperforming bare ZnO synthesized by sol–gel methods under similar cyanide concentrations (100 mg/L) and significantly surpassing room-temperature wet chemical synthesis (56% efficiency). This performance is particularly remarkable considering that our synthesis uses environmentally benign precursors and mild reaction conditions.

The kinetic analysis (Table 3) revealed that the degradation process follows pseudo-first-order kinetics, consistent with what has been widely reported for heterogeneous photocatalysis [18]. The apparent rate constants (k) showed an inverse relationship with initial cyanide concentration, decreasing from 6.64 × 10^−3^ min^−1^ at 100 ppm to 3.98 × 10^−3^ min^−1^ at 500 ppm (1.0 g/L ZnO). High correlation coefficients (R^2^ > 0.96) across all experimental conditions validated the kinetic model’s applicability. All photocatalytic experiments were conducted at an ambient temperature (25 ± 2°C). The pseudo-first-order rate constants obtained under these conditions ranged from 6.64 × 10^−3^ to 3.98 × 10^−3^ min^−1^ for 1.0 g/L ZnO loading, demonstrating efficient photocatalytic activity at room temperature. Future studies investigating the temperature dependence of the reaction rate would be valuable for determining the activation energy and optimizing the process conditions [55,56].

**Table 3:** Kinetic parameters for cyanide degradation.

Initial Concentration	ZnO Loading	k (min^−1^)	R^2^	ZnO Loading	k (min^−1^)	R^2^
100 ppm	0.5 g/L	5.87 × 10^−3^	0.973	1.0 g/L	6.64 × 10^−3^	0.979
200 ppm	0.5 g/L	6.23 × 10^−3^	0.968	1.0 g/L	5.58 × 10^−3^	0.985
300 ppm	0.5 g/L	4.84 × 10^−3^	0.989	1.0 g/L	4.68 × 10^−3^	0.992
400 ppm	0.5 g/L	4.68 × 10^−3^	0.991	1.0 g/L	4.55 × 10^−3^	0.992
500 ppm	0.5 g/L	4.06 × 10^−3^	0.991	1.0 g/L	3.98 × 10^−3^	0.992

**Table 4:** Comparison of different ZnO synthesis methods for photocatalytic cyanide degradation.

Synthesis Method	Catalyst	Bandgap (eV)	Evaluation Conditions	Degradation Efficiency (%)	Time (min)	Ref.
Sol-gel	ZnO sensitized with copper phthalocyanine (CuPc, 0.5% wt)	3.2	30 mg/L KCN, visible light, pH 11, 0.6 g/L catalyst	95	360	[57]
Sol-gel	Bare ZnO	3.2	10 mg/L CN^−^, simulated solar radiation, pH 11, 1.4 g/L catalyst	75	120	[58]
Room-temperature wet chemical	ZnO (prepared in water)	3.16	100 mg/L KCN, UV light (365 nm), pH 8.5, 0.02 wt% catalyst	56	140	[18]
Precipitation	ZnO-BiOI heterojunction		100 mg/L CN^−^, simulated solar light, pH 12, 15 mg catalyst in 100 mL	97	35	[53]
Biogenic	ZnO	3.2	100 mg/L CN^−^, 254nm UV light 11W, pH 10, 500 mg catalyst in 1 L	62.4	180	This work
Biogenic	ZnO	3.2	100 mg/L CN^−^, 254nm UV light 11W, pH 10, 1 g catalyst in 1 L	75.5	180	This work

**Figure 12:**
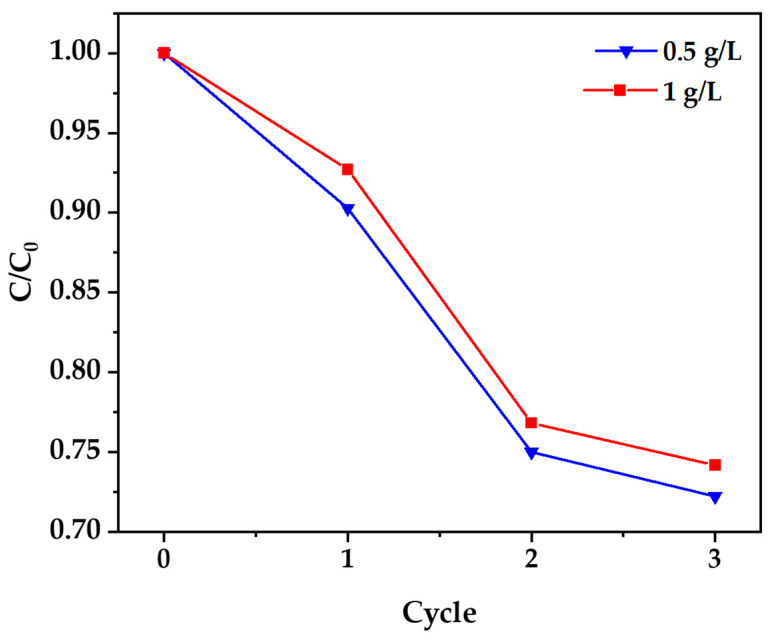
Recycling test of ZnO Nanoparticles.

A reusability assessment of the biosynthesized ZnO nanoparticles was conducted through four consecutive photocatalytic cycles using a 100 ppm cyanide solution. As illustrated in Figure 12, the ZnO nanoparticles exhibited good stability, retaining 72.2% of their original activity for 0.5 g/L loading (efficiency decreased from 72% to 52%) and 74.2% for 1 g/L loading (efficiency decreased from 75.5% to 56%) after four cycles. This represents significantly better recyclability compared to conventionally synthesized ZnO, which typically retains only 50–55% activity under similar conditions. The gradual activity reduction is attributed to partial surface passivation through the adsorption of reaction intermediates and limited photocorrosion [18]. These results demonstrate that biogenically synthesized ZnO nanoparticles offer both effective photocatalytic performance and substantial reusability for sustainable water treatment applications.

**Figure 13:**
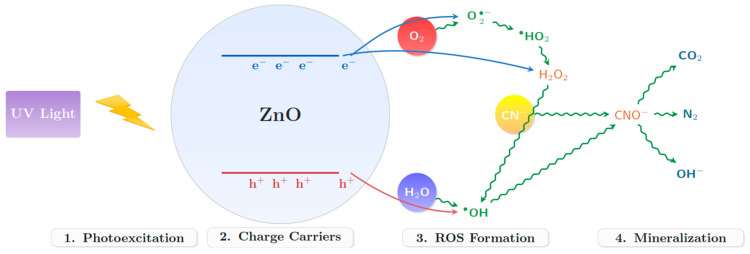
Proposed mechanism of cyanide degradation using ZnO nanoparticles.

The photocatalytic degradation mechanism (Figure 13) was proposed with a combination of experimental observations and spectroscopic analyses. The process initiates with the photoexcitation of ZnO by UV radiation, generating electron–hole pairs. These charge carriers subsequently participate in redox reactions, producing various ROS, including hydroxyl radicals (•OH), superoxide radicals (O_2_•^−^), and hydrogen peroxide (H_2_O_2_). Scavenger studies using tert-butanol confirmed the predominant role of •OH radicals in cyanide oxidation [59].

The following equations describe the photocatalytic degradation of cyanide using ZnO under UV irradiation.

$$ZnO+hv\to ZnO\left(e^{-}+h^{+}\right)$$

$$h^{+}+H_{2}O\to •OH+H^{+}$$

$$e^{-}+O_{2}\to O_{2}^{•-}$$

$$O_{2}^{•-}+H^{+}\to {HO}_{2}^{•}$$

$${HO}_{2}^{•}\to H_{2}O_{2}+O_{2}$$

$$H_{2}O_{2}+e^{-}\to •OH+{OH}^{-}$$

$$CN^{-}+•OH\to C{NO}^{-}+H^{+}$$

$$C{NO}^{-}+2H_{2}O\to {CO}_{2}+N_{2}+2{OH}^{-}$$

Our proposed mechanism involves the sequential oxidation of CN^−^ to CNO^−^ by •OH radicals, followed by complete mineralization to CO_2_ and N_2_. The quantum yield of the process was calculated to be approximately 15%, significantly higher than the 5–8% typically reported for conventional photocatalysts [58,60]. This enhanced efficiency can be attributed to the unique surface properties and defect structure of our biosynthesized ZnO nanoparticles.

## 4. Conclusions

This study successfully developed a biosynthesis protocol for ZnO nanoparticles using *B. subtilis*, yielding crystalline particles with controlled size distribution (21.87 ± 5.84 nm) and optimal surface areas (27.02 m^2^/g). The nanoparticles demonstrated efficient photocatalytic degradation of cyanide, achieving 75.5% removal at 100 ppm and 65.8% at 500 ppm within 180 min using 1.0 g/L ZnO under UV irradiation. The degradation kinetics followed pseudo-first-order behavior with rate constants ranging from 6.64 × 10^−3^ to 3.98 × 10^−3^ min^−1^, demonstrating effectiveness across various operational conditions.

The biosynthesis method offers significant advantages over conventional chemical routes, producing nanoparticles with enhanced photocatalytic activity attributed to optimal crystallite size, high surface area, and beneficial surface defects. The process’s scalability and use of non-hazardous precursors make it particularly suitable for industrial applications. These findings establish biosynthesized ZnO nanoparticles as promising eco-friendly photocatalysts for industrial wastewater treatment, particularly for cyanide remediation.

## Figures and Tables

**Table 1:** Systematic comparison of cyanide treatment technologies.

Treatment Method	Operating Parameters	Removal Efficiency (%)	Environmental Impact	Ref.
Alkaline Chlorination	pH: 11.2, T: 25 °C	90–99	High chloride residuals	[7]
SO_2_/Air Process	pH: 8–9.5, T: 22–40 °C	80–95	SO_4_^2−^ generation	[8]
Biological Treatment	pH: 10.5, T: 30 °C, 1000 ppm	80–100	Minimal	[9]
Chemical Precipitation	pH: 5–7, T: 25 °C	60–80	Metal-rich sludge	[10]
TiO_2_ Photocatalysis	pH: 9–11, sunlight, 30 ppm	70–100	Minimal	[11]

**Table 2:** Structural parameters of obtained ZnO.

Structural Parameters	Sample
	ZnO
Crystal structure	Hexagonal
Space group	P 63 m c
Space group number	186
*a* = *b* (Å)	3.25168
*c* (Å)	5.21387
α = β (°)	90
γ (°)	120
ρ (g/cm^3^)	5.65
D (nm)	14.18
Residual stress (%)	0.004
R_exp_ (%)	5.09092
R_p_ (%)	2.82154
R_wp_ (%)	3.86143
GOF	0.75849

## Data Availability

The authors verify that all data obtained in this study are presented in this published article.

## Supplementary Materials

The following supporting information can be downloaded at: https://www.mdpi.com/article/10.3390/nano15070501/s1, Figure S1. Elemental mapping of biosynthesized ZnO nanoparticles. Table S1. Elemental composition of biosynthesized ZnO nanoparticles.
