# Supplementary material for: Synthesis of ZnO Nanoparticles by Bacillus subtilis for Efficient Photocatalytic Degradation of Cyanide
Source: Nanomaterials (Basel). 2025 Mar 26;15(7):501. doi: 10.3390/nano15070501 (PMC11990931; doi:10.3390/nano15070501)
Supplement: Supplementary file 1 [file nanomaterials-15-00501-s001.zip › nanomaterials-3485790-supplementary.pdf]

Supplementary information

Synthesis of ZnO Nanoparticles by *Bacillus subtilis* for Efficient Photocatalytic Degradation of Cyanide

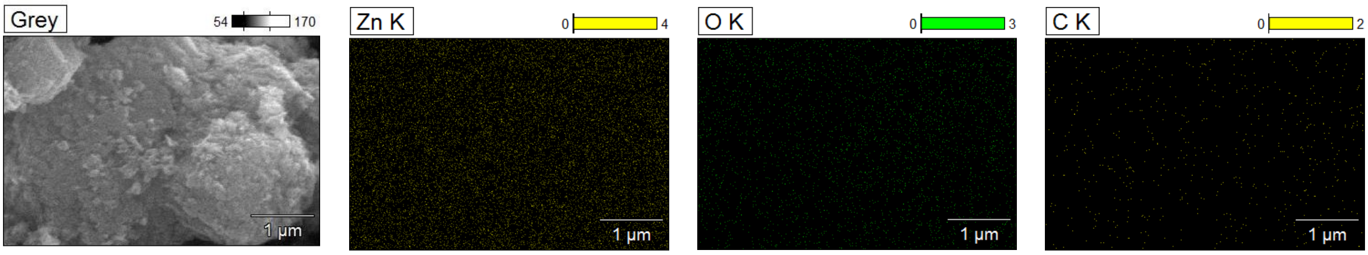

Figure S1. Elemental mapping of biosynthesized ZnO nanoparticles

Table S1. Elemental composition of biosynthesized ZnO nanoparticles

Element	Net Counts	Weight %	Atom %
**C K**	917	7.24	17.00
**O K**	6589	32.25	56.88
**Zn K**	37089	60.51	26.12
		100.00	100.00
